# Evaluation of Isoflavones as Bone Resorption Inhibitors upon Interactions with Receptor Activator of Nuclear Factor-κB Ligand (RANKL)

**DOI:** 10.3390/molecules25010206

**Published:** 2020-01-03

**Authors:** Małgorzata Zakłos-Szyda, Grażyna Budryn, Joanna Grzelczyk, Horacio Pérez-Sánchez, Dorota Żyżelewicz

**Affiliations:** 1 Institute of Molecular Biology and Industrial Biotechnology, Faculty of Biotechnology and Food Sciences, Lodz University of Technology, 90-924 Lodz, Poland; malgorzata.zaklos-szyda@p.lodz.pl; 2 Institute of Food Technology and Analysis, Faculty of Biotechnology and Food Sciences, Lodz University of Technology, 90-924 Lodz, Poland; joanna.grzelczyk@p.lodz.pl (J.G.); dorota.zyzelewicz@p.lodz.pl (D.Ż.); 3 Bioinformatics and High-Performance Computing Research Group (BIO-HPC), Computer Engineering Department, Universidad Católica de Murcia (UCAM), Guadalupe, 30107 Murcia, Spain; hperez@ucam.edu

**Keywords:** RANKL, osteoporosis, isoflavones, ITC, Saos-2, ALP, docking simulation

## Abstract

Receptor activator of nuclear factor-κB ligand (RANKL) is a cytokine responsible for bone resorption. It binds its receptor RANK, which activates osteoporosis. High levels of osteoprotegerin (OPG) competitively binding RANKL limit formation of ligand-receptor complexes and enable bone mass maintenance. The new approach to prevent osteoporosis is searching for therapeutics that can bind RANKL and support OPG function. The aim of the study was to verify the hypothesis that isoflavones can form complexes with RANKL limiting binding of the cytokine to its receptor. Interactions of five isoflavones with RANKL were investigated by isothermal titration calorimetry (ITC), by in silico docking simulation and on Saos-2 cells. Daidzein and biochanin A showed the highest affinity for RANKL. Among studied isoflavones coumestrol, formononetin and biochanin A showed the highest potential for Saos-2 mineralization and were able to regulate the expression of RANKL and OPG at the mRNA levels, as well as osteogenic differentiation markers: alkaline phosphatase (ALP), collagen type 1, and Runt-related transcription factor 2 (Runx2). Comparison of the osteogenic activities of isoflavones showed that the use of physicochemical techniques such as ITC or in silico docking are good tools for the initial selection of substances showing a specific bioactivity.

## 1. Introduction

Bone components are completely replaced within 3–10 years, taking place as a result of bone remodeling through cyclical bone formation and resorption processes. This determines the regenerative capacity of bones, such as healing injuries, wounds, and fractures. Bone resorption and formation processes are directed by two opposite cell types. Osteoblasts are bone-forming cells, while osteoclasts that are activated by osteoblasts are responsible for their destruction. Bone resorption and reconstruction must be balanced, otherwise osteoporosis or osteopetrosis will occur [1]. The signaling pathway through which osteoblasts regulate the level of activated osteoclasts involves a system of three factors: receptor activator of nuclear factor-κB ligand (RANKL)/osteoprotegerin (OPG)/receptor activator of nuclear factor-κB (RANK). RANKL occurs on the surface of osteoblasts and binds RANK found on the membrane of osteoclasts. As a result, osteoclasts differentiate into multinucleated mature forms and transform into activated cells. In that model osteoclast function is regulated by influencing actin cytoskeleton of differentiated osteoclasts [2]. Osteoprotegerin is a decoy protein that captures and binds RANKL, preventing RANK binding and osteoclast maturation. As a consequence, bone resorption is inhibited. High OPG levels protect bones from resorption [3]. Therapeutic administration of OPG to women suffering from postmenopausal osteoporosis caused a decrease in bone resorption markers by 80% [4]. RANKL/RANK signaling inhibitors are believed to play an important role in preventing bone loss. Targeting RANKL is the most effective and important approach of RANKL/RANK signaling inhibition and bone loss treatment. It has been proven that proteins with an OPG-like structure can also reduce bone resorption because they have the ability to bind RANKL as well. For this purpose, RANKL antibodies were obtained, which bound RANKL and deactivated it [5]. Peptides and peptidomimetics may also exhibit similar function [6]. A thorough understanding of the molecular mechanisms of recognition and interaction of RANKL with OPG and RANK can be helpful in assessing the ability of other substances to block RANKL.

**Figure 1:**
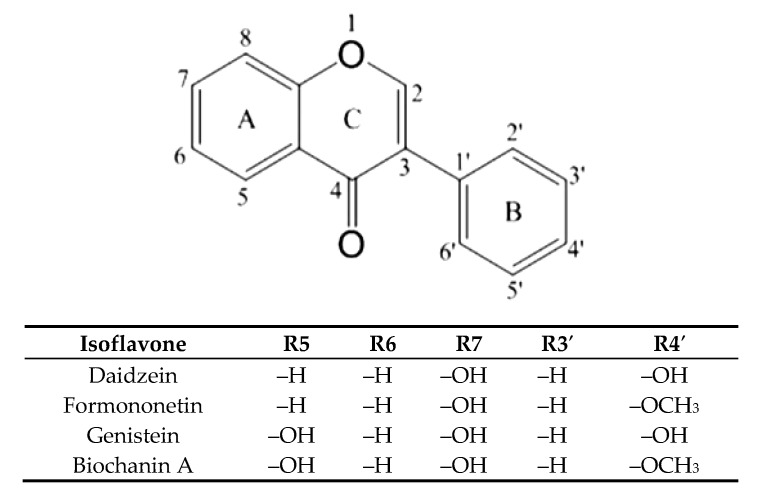
Structure of the main isoflavones.

Osteoporosis is observed in postmenopausal women, which is associated with a decrease in estrogen levels. Previous studies have confirmed the effect of isoflavones as phytoestrogens, which by binding to estrogen receptors cause an effect that reduces bone resorption. Isoflavones, by interacting with the estrogen receptor, limit interleukin 6 (IL-6) production, which stimulates osteoclastogenesis and OPG synthesis. It is also postulated osteoclastogenesis inhibition by isoflavones is a result of inactivating tyrosine kinase or involvement in cyclic AMP signaling [7]. On the other hand, blocking RANKL prevents its attachment to the receptor that inhibits osteoclast maturation and bone resorption. Animal studies are available that have shown that isoflavones reduce the excess of RANKL binding to RANK limiting bone resorption process [8]. As mentioned above, not only OPG, but also other substances, limited the effective binding of RANKL to its receptor. As many studies have shown, soy, the most commonly consumed plant legume containing mainly genistein and daidzein (Figure 1) in the form of both aglycons and glycosides, has a positive effect on bone formation. Such properties are also attributed to clover, which in turn contains mainly formononetin and biochanin A [9,10]. Also, other plant extracts, e.g., *Heimia myrtifolia* containing several phenolics, have the property of stimulating bone mineralization [11]. It has not been studied so far whether isoflavones can interact directly with RANKL as its inhibitors in the case of OPG underproduction. The aim of the study was to determine the nature of the interactions of five isoflavones found in legumes with RANKL and their affinity for the ligand, using docking simulation and isothermal titration calorimetry, and to compare the osteogenic activity using tests with Saos-2 cells, in which the degree of mineralization in the presence of tested isoflavones was checked, the level of alkaline phosphatase activity and expression of selected genes involved in bone mineralization.

## 2. Results and Discussion

### 2.1. Characteristic of Complexes of Isoflavones with RANKL

The prophylactic or therapeutic effects of dietary components can be tested using different models. The simplest are related to interactions between a biomolecule, most commonly a protein (enzyme, receptor) or nucleic acid, and a ligand, which is a component of the diet.

The biomolecule-ligand models, besides crystallographic approaches, include thermodynamic studies using the isothermal titration calorimetry (ITC) technique. This method allows for determination of a binding constant, enthalpy, and entropy of interaction, as well as free enthalpy, i.e., the affinity of a ligand to a biomolecule. There are studies available in the literature that compared the results of interactions parameters obtained using the ITC method with another model of biomolecule-ligand interaction, namely molecular modelling by docking simulation (DS) [12,13,14]. In-silico modeling allows for determination of the affinity of interactions, but additionally to the calorimetric method it indicates the site of binding [15,16,17]. Each model provides different and complementary information about the occurrence and nature of interactions.

In the studies, we determined the activity of five isoflavones against osteoporosis. Isoflavones, as shown by numerous epidemiological observations, prevent bone loss and regulate the OPG level. They may contribute to also limiting osteoporosis through other cellular mechanisms, including binding directly to RANKL, although such interactions have not been studied so far. In order to assess the potential of inactivation RANKL by isoflavones, we determined the possibility of forming complexes of isoflavone with RANKL. The interactions were evaluated at the active site where RANKL binds RANK or OPG, which compete each other.

RANKL is an approximately 35 kDa transmembrane protein with a short intracellular N-terminal tail and extracellular C-terminal region that contains the attached stalk and the receptor binding domain. Membrane-attached RANKL activates RANK to form osteoclasts through cell contact interactions. RANK is a cellular signaling surface receptor. It has a molecular weight of about 67 kDa. It is a transmembrane protein that contains four cysteine rich extracellular domains (CRDs), which are an elongation of the intracellular C-terminal region. CRDs consist of around 40 amino acid residues and contain sulfide bridges. Binding of RANKL to CRDs of RANK causes receptor trimerization and activation of osteoclastogenic signaling. OPG is a decoy receptor for RANKL with a molar mass of about 55 kDa in a form of soluble homodimer. As with RANK, the N-terminal part of OPG consists of four CRDs that bind to RANKL. Nelson, Warren, Wang, Teitelbaum, Fremont et al. [18] showed a similarity of OPG and RANK in 34%. They therefore have low sequence similarity, although folding similarity is observed. For binding of RANKL with RANK 19, amino acids of the cytokine are responsible, and with OPG 15 side residues, which are for the most part different amino acids.

**Figure 2:**
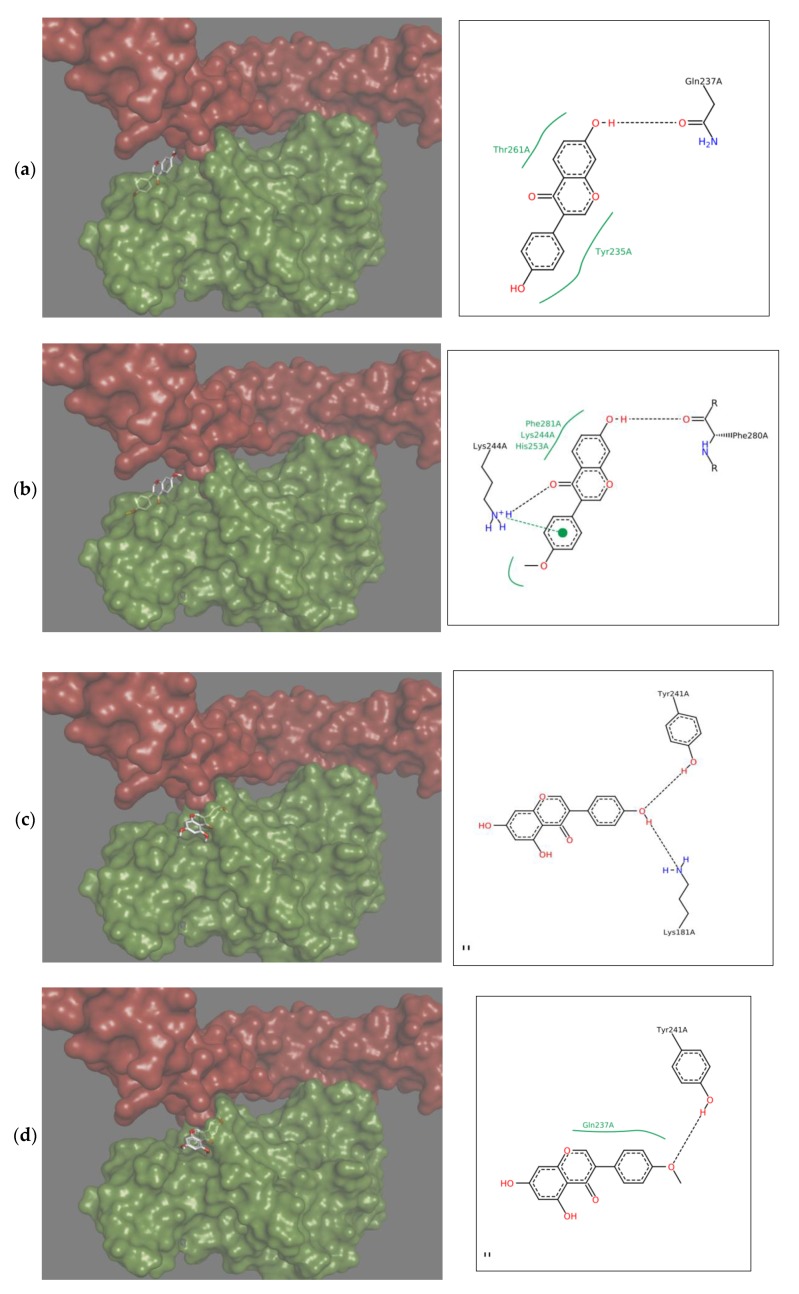

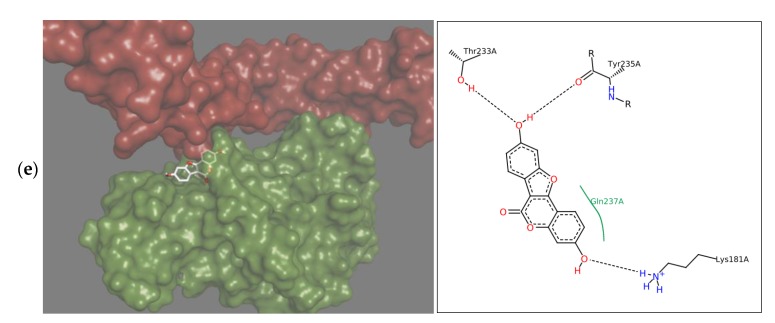
Depiction in 3D on the right (green Receptor activator of nuclear factor-κB ligand (RANKL), magenta osteoprotegerin) and in 2D on the left of the main interactions established between the active site of RANKL and: (**a**) Daidzein; (**b**) Formononetin; (**c**) Genistein; (**d**) Biochanin A; (**e**) Coumestrol. Continuous lines represent hydrophobic interactions, while black dashed lines show hydrogen bonds and dashed green lines π-π interactions.

Therapeutics that could be used in the case of insufficient OPG production should limit the binding of RANKL to RANK, and therefore the interactions should occur within the RANKL residues responsible for the formation of complex with this receptor. Docking simulation used in the study showed that each of the tested isoflavones bound RANKL within the side residues involved in RANK binding, showing the high potential of isoflavones to block the formation of such complexes. In the case of daidzein, these were bonds with TYR235 and GLN237, for formononetin with HIS235, for genistein with LYS181, and TYR241 for biochanin A with GLN237 and TYR 241, and for coumestrol three side residues located within the binding site: LYS181, TYR235 and GLN237 were engaged (Figure 2a–d). The lack of the hydroxyl group at R5 in daidzein contributed to hydrophobic interactions at TYR235 and TYR261 and in formononetin at LYS244, HIS253, and PHE281, while in biochanin A containing hydroxyl group hydrophobic interactions occurred only at one residue, i.e., GLN237. The molecular mechanism of the activation includes trimerization of the cytoplasmic tail of RANK. As demonstrated by Warren, Zou, Decker, Rohadki, Nelson et al. [3] RANK must attach to RANKL at all three binding sites to transmit the signaling. Therefore, interacting of isoflavones with RANKL even at one binding site can effectively prevent such complexing of the cytokine to the receptor and inhibit activation of osteoclasts.

Formononetin is a methoxy derivative of daidzein, and biochanin A of genistein. Methoxy forms are of high importance because they are directly and relatively quickly absorbed from the gastrointestinal tract [19]. The presence of a methoxy group resulted in an increase of hydrophobic interactions with the cytokine within the B ring of isoflavone (Figure 2a–d). On the other hand, genistein and biochanin A in relation to daidzein and formononetin have an additional hydroxyl group at R5 in the A ring (Figure 2c,d). Docking simulation showed that the presence of this hydroxyl group led to a decrease in hydrophobic interactions around the A ring of isoflavones. Xiao and Kai [20] noted that hydroxyl group in the A ring of flavonoids may attenuate hydrophobic interactions and our studies confirmed this effect among investigated isoflavones. The 3D depiction in Figure 2 showed that depending on the presence of an additional hydroxyl group in A ring, the isoflavone molecule is differently oriented in relation to the cytokine. Daidzein and formononetin, which have only one –OH group in this area, attach more parallel to the RANKL axis, where the A and C rings are directed to the center of the protein, using more hydrophobic interactions. In contrast, genistein and biochanin A attach more perpendicularly, and the hydrophobic interaction of the fragment comprising the A and C rings is weaker. The studies evaluated the effect of one coumestan, i.e., coumestrol, containing four rings. For this compound, hydrogen bonds formation was observed on two opposite sides of the molecule (Figure 2e). According to Mitchell et al. [21] coumestrol has a highly-extended π-electron system throughout the molecule via the occurrence of furan like ring D and double-bond of C-D rings, which enables conjugation to occur between the A and B rings. This may explain its easier deprotonation and hydrogen bond formation relative to daidzein and genistein, which also have two –OH groups at opposite sides of the molecules.

### 2.2. Energetic Effects of RANKL-Isoflavones Interactions

**Figure 3:**
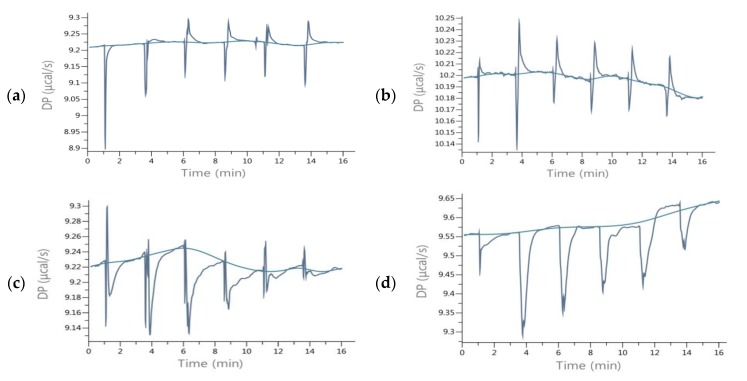
ITC raw data of titration of RANKL with isoflavones (**a**) Daidzein; (**b**) Biochanin A; (**c**) Formononetin+Biochanin A; (**d**) OPG.

The obtained enthalpies of binding of the RANKL and isoflavones indicated a different nature of interactions that yielded both exo- and endothermic effects (Table 1). It could be noticed in Figure 3a,b that during titration strong endothermic effects occurred which were the consequence of conformational changes. The total energetic effects of interactions of RANKL with most of the investigated phenolics were endothermic (Δ*H* > 0), as the exothermic interactions resulting from binding the isoflavones were weak enough to prevail energy consumption due to endothermic rearrangement effects. According to Frazier, Papadopoulou and Green [22], as well as Du, Li, Xia, Ai, Liang, Sang, et al. [23], this endothermic effects may be a result of conformational rearrangement in the protein by disruptions of the energetically favorable noncovalent interactions. As was shown by Nelsen et al. [18], the interactions of OPG or RANK with RANKL include rearrangement of the RANKL, since binding sites occur within flexible loops that rotate to match emerging energy systems. Flat flavonoids, including isoflavones, force adaptation of RANKL and cause conformational changes to form hydrogen bonds. LYS181 is a part of the A-A′ loop, while TYR235 is in the C-D loop, and interactions with these side residues were observed during DS of most of the isoflavones tested (Figure 3). In the case of titration of RANKL with isoflavones pairs, the exothermic nature of complex formation was observed, except daidzein+genistein. High exothermic effects were shown by RANKL titration with pars including the isoflavone and its methoxyl derivative, as daidzein+formononetin and genistein+biochanin A (Table 1). It confirms that, besides hydroxyl group at R5, the presence of methoxy moiety forces different type of interaction from those of non-methoxy derivative, using different side residues, and despite the structural similarity, the pair as a whole more effectively binds RANKL, and the titration curve was similar to that of OPG (Figure 3c,d).

The affinity, i.e., the change of free enthalpy during RANKL-isoflavone titration determined by ITC (Δ*G*) was calculated from Δ*H* and Δ*S* values and compared with the affinity calculated by DS (Δ*G_predicted_*) (Table 1). In both methods, the affinity was negative, which indicates the spontaneous nature of the interactions. The free enthalpy determined by the ITC method was in the range from −15.74 to −22.82 kJ/mol, for coumestrol and daidzein, respectively, and the absolute value increased in the following order: coumestrol < biochanin A < genistein = formononetin < daidzein. The affinity of isoflavones for the cytokine decreased with the presence of a hydroxyl group at R5 and a methoxy group at R4′. The stability of the complexes measured as binding constant (*K_A_*) increased in the same order (Table 1). Given the above results, daidzein may have the greatest biological significance. DS more closely reflects the static effects resulting from the docking of ligands, while the ITC also shows the energetic effects arising from protein structure rearrangement. In the case of the DS method, the values of affinity obtained did not differ to a high extent and were in the range from −23.23 to −25.53 kJ/mol for formononetin and biochanin A, respectively.

**Figure 4:**
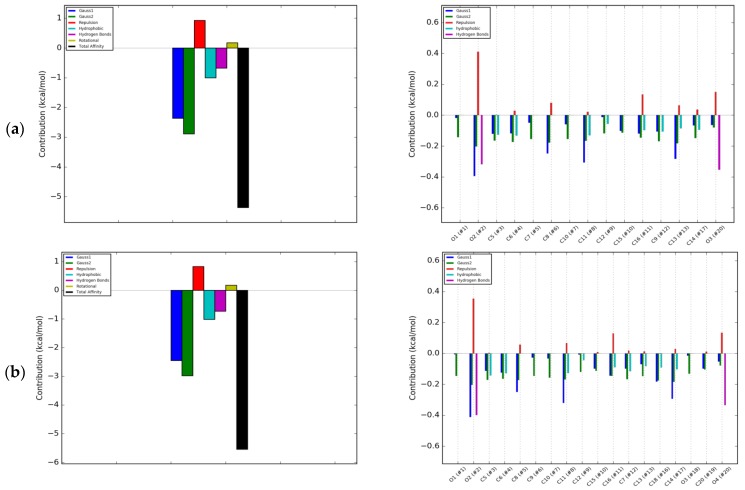

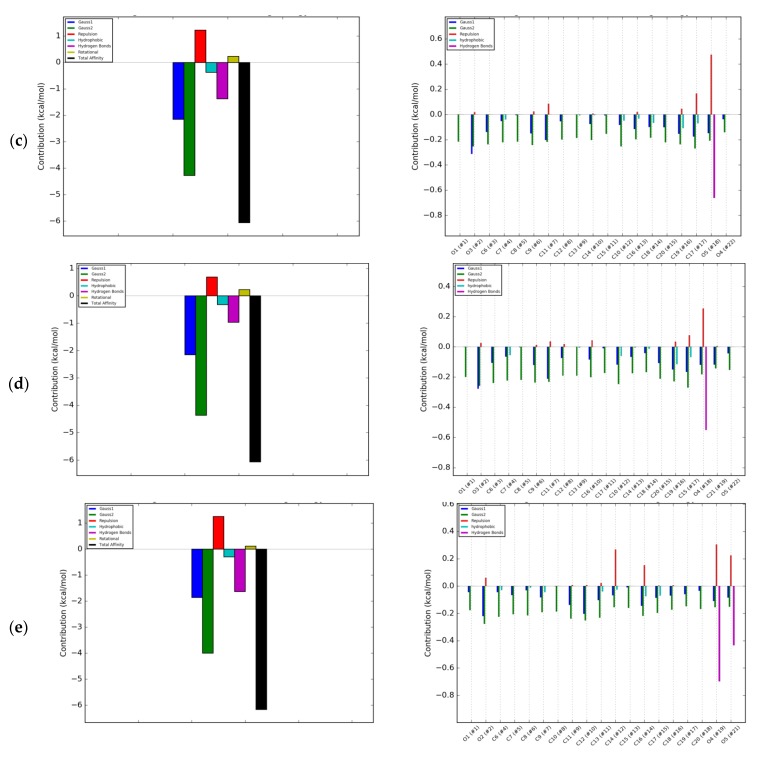
Energetic contributions to binding energy of RANKL and: (**a**) Daidzein; (**b**) Formononetin; (**c**) Genistein; (**d**) Biochanin A; (**e**) Coumestrol; blue bar—Gauss 1 interactions; green—Gauss 2 interactions; red—Repulsion; light blue—Hydrophobic interaction; magenta—Hydrogen bonds; olive—Rotational; back—Total affinity.

In the DS model, isoflavones lacking a hydroxyl group at the A ring, i.e., daidzein and formononetin, were characterized by a higher energy of hydrophobic interactions than of hydrogen bonds (Figure 4a,b). In contrast, genistein and biochanin A exhibited significantly higher hydrogen binding interactions compared to hydrophobic ones (Figure 4c,d). Similar energy effects were also observed with coumestrol (Figure 4e). Hydrogen bonds occurred mainly at the oxygen atom O4′ in the B ring, and their formation caused a repulsive effect, weakening the overall effect of energetic affinity. The energetic effect associated with the rotation and tension of bonds also contributed to the weakening of the interactions, but for all the investigated isoflavones it was of marginal significance. The largest isoflavone tensile effects associated with fitting of the molecule were observed for biochanin A. The greatest impact for the interactions of the RANKL cytokine with isoflavones had electrostatic interactions described as Gauss 1 and Gauss 2, which were not concentrated at the selected regions of isoflavone; hence, they were not marked on the 2D projection. Due to the high contribution of electrostatic interactions to Δ*G_predicted_*, they determined the overall negative value of free enthalpy and the spontaneous nature of the interaction. RANKL interactions with RANK are of electrostatic type compared to those with OPG [3]. The conclusion is that isoflavones such as formononetin, biochanine A, and coumestrol interact with RANKL more similarly to RANK than OPG, since the nature of their interaction is predominantly electrostatic.

In the evaluated interactions of the RANKL with pairs of isoflavones, the resultant affinity was in the range from −15.07 kJ/mol for daidzein with biochanin A to −36.22 kJ/mol for formononetin with biochanin A (Table 1). The curve of titration of RANKL with solution of formononetin +biochanin A was the most similar to that of titration with OPG solution (Figure 3c,d), indicating that these two isoflavones can act in common as an effective RANKL decoy. The pair of methoxy isoflavones may be a good candidate to develop preparations for use as osteoporosis therapeutics, showing that plant extract containing a complex mixture of few isoflavones may act as a very effective therapeutic in contrast to one isolated substance. Intake of biochanin A in foods is additionally supported by the fact that biochanin A blood level has been shown to be higher when consumed in the presence of other isoflavones [24]. Intake of isoflavones may be particularly beneficial in preventing osteoporosis when they are additionally accompanied by proteins of both plant and animal origin [25]. Numerous previous studies have indicated that soybean, the most commonly consumed legume rich in phytoestrogens such as genistein and daidzein glycosides, has osteogenic activity. Less often, such properties were attributed to clover, which in turn contains mainly formononetin and biochanin A [9]. Studies by Singh et al. [10] confirmed the regenerative effect of formononetin on bone. Our research has shown that methoxy derivatives of isoflavones are more active in interactions with RANKL, and hence clover preparations may be more effective against osteoporosis than soy. In addition, Su et al. [9] stated that genistein is a weak selective as an estrogen receptor activator and activates both the α and β forms, and intake of clover preparations is more favorable compared to soy preparations. The α form activation may be responsible for the proliferation of breast and uterus cancer, while biochanin A is a selective β estrogen receptor activator and does not activate cancerous processes in these organs. So far, the osteogenic effect of isoflavones has been equated with the activation of estrogen receptors [8]. In our research, we have shown a possible other mechanism to prevent osteoporosis.

### 2.3. Effect of Isoflavones on Saos-2 Viability and Mineralization

**Figure 5:**
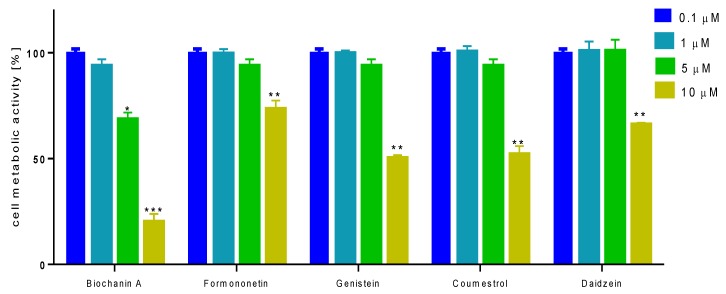
The influence of isoflavones on Saos-2 cells metabolic activity determined by PrestoBlue assay after 14 days exposure. Control cells were not exposed to any compound but the vehicle; values are means ± standard deviations from at least three independent experiments (*n* ≥ 16); statistical significance was calculated versus control cells (untreated); * *p* ≥ 0.05, ** *p* ≥ 0.01, *** *p* ≥ 0.001.

To check the influence of selected isoflavones on bone mineralization process under in vitro conditions we used human osteosarcoma Saos-2 cell line, which exhibits osteoblastic properties with high levels of alkaline phosphatase (ALP) activity [26,27]. Due to the fact that in the presence of mineralizing conditions cells undergo differentiation to osteocytes in terms of morphology and genes expression, first we studied the effect of isoflavones concentrations from 0.1 to 10 µM on cellular viability after 14 days incubation. As is presented in Figure 5, the metabolic activity decreased with increasing compounds concentrations starting from 1–2.5 µM. The highest influence on metabolic activity revealed biochanin A, the lowest influence on cells viability showed formononetin. The IC_50_ values, described as the chemical concentration required to reduce cellular activity to 50% of the control, are summarized in Table 2. According to IC_50_ values, the cytotoxicity of studied isoflavones ranked as follows: biochanin A > genistein > coumestrol > daidzein > formononetin.

To compare biological activity of phenolics as the highest noncytotoxic concentration (IC_0_) selected for further studies with cells 1 µM was chosen. Earlier clinical studies have shown, depending on the experimental conditions, that soy isoflavones in the 40–110 mg daily range are effective in inhibiting bone resorption [28]; however, the maximum recommended level for safe isoflavone intake is set at 75 mg/day, and a plasma concentration does not exceed 10 µM [29,30]. In further experiments we used 1 µM dosage resulted from viability test, which according to Su et al. [9] corresponds to the consumption of about 7.5 mg per day, so it remains in a safe range.

**Figure 6:**
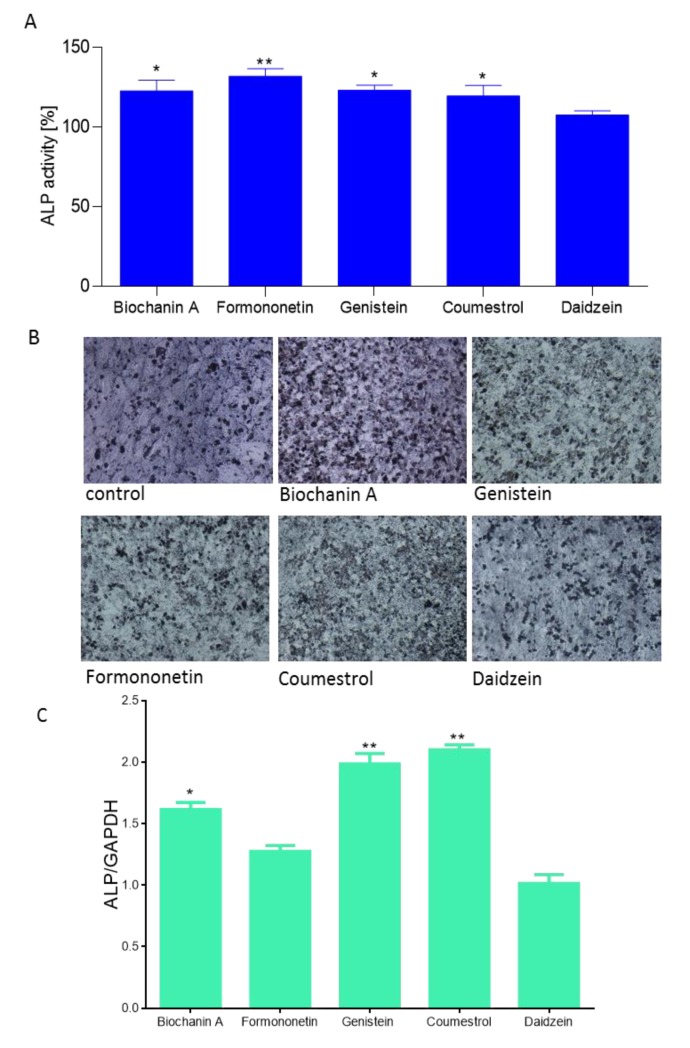
The influence of isoflavones on Saos-2 cells alkaline phosphatase (ALP) activity determined by *p*-nitrophenyl phosphate (*p*NPP) assay after 14 days exposure. Control cells were not exposed to any compound but the vehicle; values are means ± standard deviations from at least three independent experiments (*n* ≥ 15) (**A**); representative images of cells incubated in the presence of ALP substrate BCIP/NBT after treatment with isoflavones using Nikon TS100 Eclipse microscope (Japan), 200× magnification (**B**); isoflavones as regulators of ALP expression gene quantified by real-time PCR and normalized using glyceraldehyde-3-phosphate dehydrogenase (GAPDH) as a reference gene (*n* ≥ 3); (**C**); statistical significance was calculated versus control cells (untreated), statistical significance was calculated versus control cells (untreated), * *p* ≥ 0.05, ** *p* ≥ 0.01, *** *p* ≥ 0.001.

Due to the fact that isoflavones have been demonstrated as stimulators of osteogenesis [31], in a further step we checked their influence on an early marker of osteoblast differentiation, essential for mineralization ALP enzyme activity. As is shown in Figure 6A, all studied compounds at 1 µM dosage after 14 days incubation were able to increase ALP activity between 5 to 29%. Daidzein was identified as the weakest ALP activator, whereas formononetin was the strongest. 

Cells incubation in the presence of ALP substrate BCIP/NBT allowed their staining and imaging with bright-field microscopy (Figure 6B). Increased ALP activity correlated with *ALP* gene expression elevation at the transcriptional level (Figure 6C). Saos-2 cells treated with biochanin A, genistein and coumestrol demonstrated a 1.6, 1.9 and 2.1-fold increase in *ALP* mRNA expression relative to vehicle-treated control cells (normalized to 1.0), respectively. Formononetin and daidzein had no influence on the *ALP* mRNA expression level. Daidzein seemed to have slight influence on ALP protein activity, as well as its transcriptional level. Contrary results obtained for formononetin suggest that in the presence of formononetin enhanced activity of ALP protein results from post-transcriptional, post-translational or potential epigenetic mechanisms.

**Figure 7:**
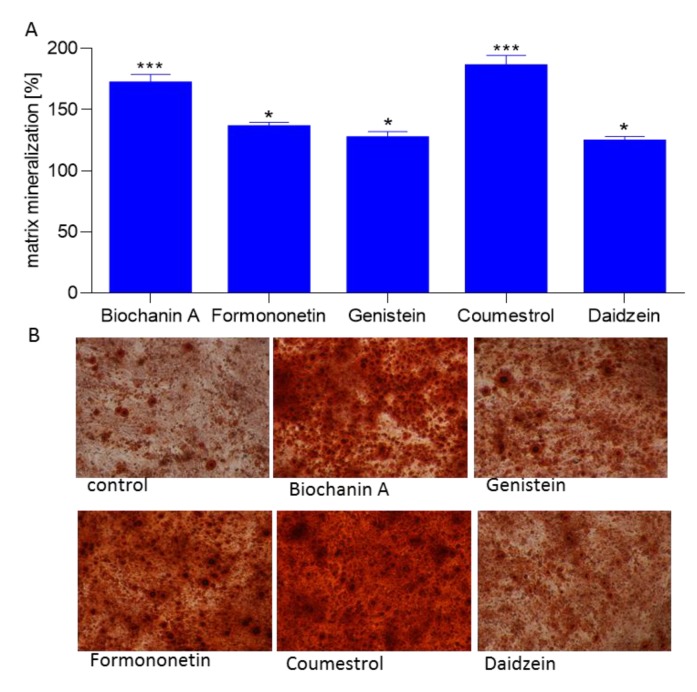
The influence of isoflavones on Saos-2 cells matrix mineralization determined by Alizarin Red S spectrophotometric assay after 14 days exposure. Control cells were not exposed to any compound but the vehicle; values are means ± standard deviations from at least three independent experiments (*n* ≥ 9) statistical significance was calculated versus control cells (untreated), * *p* ≥ 0.05, ** *p* ≥ 0.01, *** *p* ≥ 0.001 (**A**); representative images of cells stained with Alizarin Red S after treatment with isoflavones using Nikon TS100 Eclipse microscope (Japan), 200× magnification (**B**).

Further, the ability of isoflavones to generate calcified extracellular matrix was confirmed by Alizarin Red S staining quantification. All studied phenolics were able to stimulate osteogenic differentiation through production of calcified extracellular matrix between 21% to 87% (Figure 7A). The strongest biomineralization activity showed coumestrol and biochanin A which significantly increased the extent of mineral deposition to almost 1.8 and 1.7-fold compared with control cells, respectively. Formononetin, genistein, and daidzein exposed comparable influence on biomineralization. That quantitative result has been confirmed by bright-field microscopic observations: the cellular calcified matrix is characterized by round-shaped granules stained in red (Figure 7B). 

**Figure 8:**
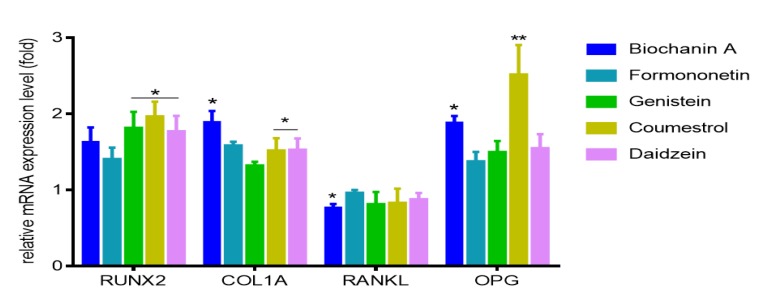
The influence of 14 days of exposure of isoflavones on the expression of genes in Saos-2 cells. The expression levels of *RUNX2*, *COL1A*, *RANKL*, *OPG* were quantified by real-time PCR and normalized using glyceraldehyde-3-phosphate dehydrogenase (*GAPDH*) as a reference gene. Control cells were not exposed to any compound but the vehicle; values are means ± standard deviations, *n* ≥ 3; statistical significance was calculated versus control cells (untreated), * *p* ≥ 0.05, ** *p* ≥ 0.01.

In cells treated with coumestrol cultures were heavily mineralized with cell monolayers completely covered with mineralized matrix, a process resulting from intracellular accumulation of the mineralizing ions like HCO_3_^−^, CO_3_^2−^, PO_4_^3−^, and Ca^2+^ [26]. The process of bone formation is related with increased ALP activity and mineralization; nevertheless, type 1 collagen, being the main bone matrix structural protein, plays a very important role in that process. Thus, we examined the expression of osteoblast marker gene: *COL1A1* (collagen type 1) in differentiated Saos-2. As is presented in Figure 8, all isoflavones increased *COL1A1* expression level from 35% to 89%, where the maximum stimulatory effect was observed for biochanin A. However, bone associated genes coding studied by us ALP (alkaline phosphatase), type-I collage or osteocalcin, are regulated by transcription factor Runx2 (Runt-related transcription factor 2) known as the key osteoblast differentiation regulator. Runx2 binds to OSE element (osteoblast specific *cis* acting element) in the promoter regions of major osteoblast specific genes and control their expression [31].

Therefore, next we intended to study the influence of isoflavones on Runx2 expression level. All studied compounds were able to increase by at least 50% its mRNA expression level, whilst it was especially pronounced after cells incubation with coumestrol (Figure 8). Taking into account obtained results we can conclude that Runx2 abundance increase is followed by elevation of Runx2 protein expression, its transcriptional activity enhancement, and induction of functional activity of osteoblasts via matrix mineralization.

Our previous results with ITC and docking simulation showed that isoflavones can form complexes with RANKL limiting its binding to RANK receptor. The balance of surface located RANKL and OPG acting as decoy soluble receptor for RANKL, plays crucial role in balance of bone remodeling [18]. Due to observed amplified mineralization of Saos-2 extracellular matrix, we therefore studied isoflavones possible influence on expression of *RANKL* and *OPG* in differentiated cells. Among the studied phenolics, only biochanin A downregulated mRNA level of RANKL expression by almost 20%, while other compounds had no significant influence in that field (Figure 8). However all of them increased *OPG* at the transcriptional level. The most prominent increase in the mRNA expression of osteoprotegerin was detected for coumestrol and biochanin A with 1.8 and 2.4-fold, respectively. Compounds decreasing RANKL/OPG mRNA expression ratio are described as reducers of osteoclastic cell recruitment, thus playing an important role in the downregulation of osteoclastic cell activities [32]. According to these calculations, RANKL/OPG ratios were reduced upon cells treatment with biochanin A and coumestrol with values equal to 39% and 33% (comparing to normalized control), respectively. Thus, we can assume that the main isoflavones present in red clover or chickpea may act as mineralization enhancers through complexing RANKL, but also via up regulation of ALP activity and expression levels of mRNA coding Runx2 and OPG, as well as down regulation of RANKL/OPG ratio in differentiated Saos-2 cells.

There is data confirming positive effects of different polyphenols on the mineralization process [31]. Studies performed on rat primary adipose-derived stem cells (ADSCs) showed that biochanin A increased cellular mineralization and ALP activity, as well as stimulated the expression of the ALP, osteocalcin, Runx2, and OPG [33]. Further studies performed with the ovariectomized rat model of osteoporosis confirmed its osteoblastic activities through an increase of mRNA expression levels of osterix, collagen type I, alkaline phosphatase, and osteocalcin, which were followed by decreased RANKL/OPG ratio [9]. Genistein was also shown as phytocompound able to elevate alkaline phosphatase activity and decrease RANKL/OPG ratio simultaneously in Saos-2 [32]. However, there is data matching positive activity of isoflavones, mainly genistein, with binding properties to estrogen receptors expressed by osteoblasts during bone mineralization in MG-63 cells [34]. Experiments performed on murine and rat osteoblastic cells (MC3T3-E1 and UMR-106) showed that genistein displayed osteoblastic activities through its higher binding affinity toward ERβ leading to increased ALP and OPG levels, and reduced RANKL [35,36].

Research performed on genistein treated osteoporotic, ovariectomized rats additionally presented the increase of breaking strength and bone quality. Osteoblast differentiation also involves daidzein, which was able to promote cell viability, ALP activity, and collagen type 1 production in human osteoblast-like MG-63 and osteoblastic OCT1 cells [37,38]. In observed processes there was detected not only an elevation of bone morphogenetic protein BMP2, one of the member of transforming growth factor-β superfamily synthesized and secreted by osteoblasts, but also estrogen receptors signaling mitogen-activated protein kinases/extracellular signal-regulated kinases (MAPK/ERK) and phosphoinositide-3-kinase/serine-threonine kinase (PI3K/Akt) involvement. Studies comparing activities of formononetin, genistein, and daidzein performed on Saos-2 cells and rats with ovariectomy-induced bone loss in rats proved that all compounds prevented bone loss, yet formononetin was the strongest alkaline phosphatase activator, genistein inhibited osteoclast proliferation most efficiently, daidzein didn′t significantly inhibit osteoclast proliferation and had no influence on ALP activity [38]. Results obtained from studies performed on human normal osteoblasts showed that formononetin was able to increase osteogenic markers levels, including BMP2 [39]. Mineralization potential was also demonstrated for coumestrol, which was able to increase ALP activity and its gene expression, type I collagen gene expression, and osteoprotegerin secretion by neonatal and adult mice osteoblasts [40].

Taken together, our results showed mineralization potential of the main isoflavones identified of legumes: biochanin A, formononetin, genistein, coumestrol, and daidzein. Among studied isoflavones coumestrol, formononetin, and biochanin A showed the highest potential for Saos-2 mineralization and were able to regulate the expression of RANKL and OPG at the mRNA levels, as well as osteogenic differentiation markers: alkaline phosphatase, collagen type 1, and Runt-related transcription factor 2 (Runx2).

Molecular mechanism of phytoestrogens influence on bone mineralization is very complex and there are many possible cellular pathways that can be involved in observed mineralization of Saos-2 cells. The reaction between released RANKL and isoflavones via its binding is very relevant in a situation where osteoclasts are involved: downregulation of free RANKL protein will lower its binding to RANK receptor, decrease commitment of monocyte/macrophage precursor cells to the osteoclast lineage, and, finally, the activation of mature osteoclasts involved in bone resorption [41]. The observed enhanced Saos-2 mineralization is the result of various cellular answers generated via different types of stimuli, not only reduction of RANKL/OPG. 

To the fact that identification of all aspects of molecular mechanisms of isoflavones present in legumes influence on mineralization is quite complicated, for the beginning we chose isoflavones interactions with RANKL and their possible influence on functional mineralization process in Saos-2 cells. The mechanism of isoflavones effect on osteoclasts probably does not depend on estrogen mechanisms, as there are no ERs in the osteoclast nuclei [25]. Presented results are necessary and basal for our further studies and they do not exclude involvement of different types of signaling transduction pathways. The effects of soy intake in various preparations, mainly containing genistein and daidzein, on inhibiting bone resorption have been intensively studied. Many clinical studies involving a six-month intake of soy preparations showed that in many cases a beneficial effect on bone mass was observed. However, it might be influenced by the period of isoflavones intake, the composition of the diet, and individual features. Other plants containing isoflavones, mainly in the form of methoxy derivatives such as chickpeas and red clover, do not have such a large research history.

## 3. Materials and Methods 

### 3.1. Chemicals and Reagents

OPG recombinant, expressed in *E. coli* ≥ 98%, sRANKL recombinant, expressed in *E. coli* ≥ 98%, daidzein ≥ 98%, biochanin A ≥ 95%, genistein ≥ 98% formononetin ≥ 99% coumestrol ≥ 97.5% and ITC reagents of LC/MS grade were obtained from Sigma Aldrich (St. Louis, MO, USA). All cell culture reagents were obtained from Life Technologies (Carlsbad, CA, USA). Tissue culture plastics were supplied by Greiner Bio-One GmbH (Frickenhausen, Austria). All the experimental measurements, if not stated otherwise, were performed using the Synergy 2 BioTek microplate reader (Winooski, VT, USA). Microscopic observations were performed using fluorescent microscope Nikon TS100 Eclipse (Tokyo, Japan) under 200× magnification if not stated otherwise.

### 3.2. Isothermal Titration Calorimetry

Calorimetric measurements were conducted using a MicroCal PEAQ-ITC200 (Malvern, Worcestershire, UK) isothermal titration calorimeter. Analyses were carried out according to Budryn et al. [15], with some modifications. We had to use very dilute solutions and decided to conduct the analysis with fewer injections and increased titrant volume. The calorimeter cell (0.2 mL) was filled with degassed 0.1 μM RANKL solution and 12 μL portions of degassed 10 μM aqueous solutions of isoflavones or OPG were injected into the cell. Measurements were carried out at 25 °C with continuous stirring (307 rpm). Isoflavone or OPG aliquots were injected at intervals depending on the time value of the observed effects. Measurements of the thermodynamic effects of dilution of titrants solutions were additionally carried out to subtract those effects from the RANKL titration measurements. The effects of dilution of the RANKL solution was negligible.

The analysis was carried out for individual isoflavones or pairs of them to capture synergistic effects at concentrations of 5 μM of each substance. The heat released upon interactions between RANKL and the isoflavone was recorded over time, and the raw data were obtained as a plot of heat flow in kcal/s against time. The integration of each peak yielded a value for the heat released during each injection of the isoflavone solution into the cell filled with RANKL solution. The plot of these values vs. the molar ratio titrant/RANKL was then used to determine the thermodynamic parameters of interactions. Parameters such as binding constant (*K_A_*), enthalpy change (Δ*H*), and entropy change (Δ*S*) were calculated from the ITC titration nonlinear least-squares curve fitting carried out in MicroCal PEAQ-ITC200 software using single binding mode. The free energy change (Δ*G)* was calculated from the Gibbs equation [17].

### 3.3. Molecular Modeling

In order to obtain detailed information at the atomic level about the interactions between the different isoflavones and RANKL, we carried out molecular modeling studies. We performed docking simulations to provide information on how hydrogen bonds and hydrophobic, as well as other types of interactions between bioactive compounds and RANKL are formed [18].

A representative X-ray crystal structure for human RANKL/OPG complex was 3URF taken from the Protein Data Bank (PDB) database (http://www.rcsb.org/structure). Next, a full atom model of the protein was prepared from the PDB structure. Bond orders were assigned, hydrogens were added, and cap termini were included with the Protein Preparation Wizard module available in Maestro [37]. The protonation states of all side chains were subsequently defined using PROPKA3.1. The chemical structures of all isoflavone molecules were built up manually and partial charges were calculated using Gasteiger scheme to be used during docking simulations [42].

The docking of isoflavones to the prepared RANKL/OPG structure model was performed with Autodock Vina docking software [43] using default configuration parameters. The size of the grid box for ligand docking was set to extend 60 A° in each direction from the geometric centre of each individual docking simulation. The scoring function in Vina considers the Lennard-Jones term (LJ), hydrogen bonds (H-bonds), electrostatic interactions, hydrophobic stabilization, entropic penalty due to the number of rotatable bonds, and the internal energy of the ligand.

### 3.4. Cell Culture

Studies were performed with Saos-2 cell line-human osteosarcoma cells obtained from American Type Culture Collection (Manassas, VA, USA), which displays several osteoblastic features. Cells were cultured in DMEM-low glucose with 10% fetal bovine serum (FBS) medium supplemented with 100 U/mL penicillin, 100 μg/mL streptomycin and 25 μg/mL amphotericin B. After 24 h osteogenesis process was induced with medium consisting of DMEM-low glucose completed with 2-phospho-l-ascorbic acid (100 μM), L-proline (34.8 μM) and β2- glycerol phosphate (5 mM) [44]. On the following day, medium was changed with fresh osteogenic medium containing isoflavones for 14 days. Medium was changed every two days. Cells were maintained at 37 °C in a humidified incubator containing 5% CO_2_. Tested compounds were dissolved in a PBS/DMSO (1:1 *v*/*v*) at concentrations used in biological studies presented in the descriptions of the tests carried out.

### 3.5. Cell Viability

Cells were seeded in complete medium and grown for 24 h, then medium was changed into osteogenic medium and cells were incubated in the presence of the studied extracts diluted in culture medium for 96 h. Cells viability was quantified with PrestoBlue reagent according to the manufacturer′s instructions by measuring the fluorescent signal at F530/590 nm. The obtained fluorescence values were used to calculate cell viability expressed as the percentage of the viability of the untreated control cells (cells treated with equal volume of the vehicle instead of the preparation).

### 3.6. Alizarin Red Cells Staining

Mineralization on the matrix synthesized by the monolayer of Saos-2 cells was analyzed with alizarin red staining according to Muthusami et al. [45] with modifications. Briefly, after 14 days, cells were washed with PBS and fixed by the adding of 5% formaldehyde solution for 30 min at room temperature. After rinsing with water, 1% alizarin red S in 2% ethanol (pH 4.0) was added and allowed to stand for 30 min at room temperature with gentle shaking. The monolayers were rinsed 5 times with destilled water and cells were observed under microscope. To quantify matrix mineralization, cells were incubated with 100 mM cetylpyridinium chloride for 1 h and gentle shaked to solubilize and release calcium-bound alizarin red S into solution and absorbance at 570 nm was measured. The obtained absorbance values were used to calculate matrix mineralization as the percentage of the absorbance of the untreated control cells.

### 3.7. Estimation of ALP Activity

After 14 days cells were rinsed with PBS, then 1.0 mg/mL *p*-nitrophenyl phosphate (*p*NPP) in 0.2 M Tris buffer as the substrate for alkaline phosphatase was added [45]. After 15 min of incubation at 37 °C absorbance at 405 nm was measured. For cells visualization of ALP activity under microscope BCIP (5-bromo-4-chloro-3-indolyl phosphate)/ NBT (nitroblue tetrazolium) substrate was added.

### 3.8. Gene Expresssion Analysis

Total RNA was extracted from cell culture after 14 days of incubation with compounds in osteogenic medium using GeneMatrix Universal RNA Purification Kit (Eurex Ltd., Gdansk, Poland) according to the manufacturer′s procedure. RNA samples were purified with Amplification Grade DNase I and reverse transcribed with NG dART RT Kit (Eurex Ltd., Gdansk, Poland). Real time RT-PCR was carried out using SG qPCR Master Mix (Eurex Ltd., Gdansk, Poland) on a BioRad CFX96 qPCR System (Bio-Rad, Hercules, CA, USA). Complementary DNA representing 6 ng of total RNA per sample was subjected to 25 to 40 cycles of PCR amplification. Samples were first incubated at 95 °C for 40 s, then at 55 °C for 30 s, and finally at 72 °C for 30 s. To exclude non-specific products and primer-dimers, after the cycling protocol, a melting curve analysis was performed by maintaining the temperature at 52 °C for 2 s, followed by a gradual temperature increase to 95 °C. The threshold cycle (Ct) values for that gene did not change in independently performed experiments. The level of target gene expression level was calculated as 2−ΔΔCt, where ΔΔCt = [Ct(target) − Ct(βactin)]sample − [Ct(target) − Ct(βactin)] calibrator. Gene expression was normalized using constitutively expressed glyceraldehyde-3-phosphate dehydrogenase (*GAPDH*) as a reference gene. The following primer sequences were used to determine the genes′ expression: *RUNX2* 5′- CAGTTCCCAAGCATTTCATCC-3′ (F) and 5′-TCAATATGGTCGCCAAACAG-3′ (R); *ALP* 5′- ACCTCGTTGACACCTGGAAG-3′ (F) and 5′-CCACCATCTCGGAGAGTGAC-3′ (R); *COL1A1* 5*′-* GCCAAGACGAAGACATCCCA-3′ (F) and 5′-CACCATCATTTCCACGAGCA-3′ (R); *RANKL* 5′- GAGTTGGCCGCAGACAAGA-3′ (F) and 5′-TTGGAGATCTTGGCCCAACC-3′ (R); *OPG* 5′- CAGCGGCACATTGGAC-3′ (F) and 5′-CCCGGTAAGCTTTCCATCAA-3′ (R); *GAPDH* 5′- CCACCCATGGCAAATTCCATGGCA-3′ (F) and 5′-TCTAGACGGCAGGTCAGGTCCACC-3′ (R). Data analyses were obtained from at least three independent experiments.

### 3.9. Statistical Analysis

ITC analysis was conducted at least three times for each isoflavone and OPG solution. Statistical analysis was based on the determination of the average values of measurements and their standard deviation, as well as one-way ANOVA (analysis of variation) and t-Student test, using Statistica 10.0 software at the significance level *p* < 0.05. For biological studies determination of average values and one-way ANOVA analysis followed by the Dunnett′s test were performed using GraphPad Prism 6.0 software (GraphPad Software, Inc., La Jolla, CA, USA) at the significance level of * *p* < 0.05, ** *p* < 0.01, *** *p* < 0.001.

## 4. Conclusions

Considering the activity of isoflavones as nutraceuticals complexing RANKL helpful in the prevention of osteoporosis, it should be taken into account whether they are administered as isolated individual substances or as a mixture in functional foods. Evaluation of interactions of isoflavones with RANKL showed high affinity of daidzein and biochanin A as single substances, determined by ITC method and docking simulation. The cytokine was titrated by ITC also with pairs of isoflavones and promising results of RANKL complexing were obtained for methoxy derivatives comprising formononetin and biochanin A. We confirmed isoflavones potential for Saos-2 cells mineralization and coumestrol, formononetin, and biochanin A were the strongest regulators of the expression of RANKL and OPG at the mRNA levels, as well as osteogenic differentiation markers: alkaline phosphatase, collagen type 1 and Runt-related transcription factor 2. These phenolics are components of legumes such as red clover and chickpea and are characterized by quick absorption in the digestive tract. Soy is on a contrary a rich source of genistein and daidzein. In a subsequent stage of research, we plan to investigate the osteogenic effect of legume extracts of high concentration of methoxyl derivatives of isoflavones.

## Figures and Tables

**Table 1:** Thermodynamic parameters of interactions of isoflavones with RANKL determined by isothermal titration calorimetry (ITC) and docking simulation (∆*G_predicted_*).

Isoflavone	*K_A_* × 10^3^ (L/mol)	∆*H* (kJ/mol)	∆*S* (J/mol*K)	∆*G* (kJ/mol)	∆*G_predicted_* (kJ/mol)
Daidzein	15.95 ± 4.05	−0.05 ± 0.01 ^b^	78.94 ± 10.12 ^a^	−22.82	−23.65 ^a^
Formononetin	3.14 ± 0.72 ^a^	0.19 ± 0.04 ^a^	67.61 ± 7.92	−19.30 ^c^	−23.23 ^a^
Genistein	3.15 ± 0.59 ^a^	2.47 ± 0.48	75.69 ± 9.09 ^a^	−19.34 ^c^	−25.32 ^b^
Biochanin A	1.20 ± 0.47	22.90 ± 4.09	138.42 ± 21.28 ^b^	−17.00	−25.53 ^b^
Coumestrol	0.70 ± 0.11	18.55 ± 2.17	118.92 ± 17.11	−15.74 ^a^	−25.11 ^b^
OPG	720.15 ± 95.41	−10.80 ± 1.77	17.15 ± 2.54	−15.72 ^a^	-
Daidzein + Formononetin	-	−21.94 ± 3.54 ^e^	−19.47 ± 3.21 ^d^	−16.33	-
Daidzein + Genistein	-	21.10 ± 2.08	137.89 ± 18.03 ^b^	−18.63	-
Daidzein + Biochanin A	-	−20.05 ± 4.17 ^e^	−17.29 ± 4.19 ^d^	−15.07	-
Daidzein + Coumestrol	-	−5.74 ± 0.90 ^c^	41.25 ± 5.12	−17.63 ^b^	-
Formononetin + Genistein	-	0.05 ± 0.02 ^a,b^	76.75 ± 15.07 ^a^	−22.06	-
Formononetin + Biochanin A	-	−5.40 ± 0.82 ^c^	106.94 ± 11.34	−36.22	-
Formononetin + Coumestrol	-	−8.42 ± 1.93 ^d^	32.98 ± 4.18 ^c^	−17.92	-
Genistein + Biochanin A	-	−21.56 ± 5.73 ^e^	−20.78 ± 3.05	−15.57 ^a^	-
Genistein + Coumestrol	-	−15.37 ± 3.03	7.85 ± 1.83	−17.63 ^b^	-
Biochanin A + Coumestrol	-	−8.50 ± 2.15 ^d^	30.66 ± 5.22 ^c^	−17.33 ^b^	-

*K_A_*—Binding constant; Δ*H*—Enthalpy change; Δ*S*—Entropy change; Δ*G*—Free energy change—Total affinity; the same letters in one column indicate no significant differences (*p* > 0.05).

**Table 2:** The influence of isoflavones on Saos-2 cells viability presented as IC_50_ values after 14 days incubation. The influence of tested compounds was measured with PrestoBlue assay, values are means from at least three independent experiments (*n* ≥ 9).

Isoflavone	IC_50_ [µM]
Biochanin A	7.0 ± 0.3
Formononetin	25 ± 1.5
Genistein	10 ± 0.6
Coumestrol	12 ± 0.3
Daidzein	20 ± 1.5
